# Golf Swing Biomechanics: A Systematic Review and Methodological Recommendations for Kinematics

**DOI:** 10.3390/sports10060091

**Published:** 2022-06-09

**Authors:** Maxime Bourgain, Philippe Rouch, Olivier Rouillon, Patricia Thoreux, Christophe Sauret

**Affiliations:** 1 Arts et Metiers Institute of Technology, Université Sorbonne Paris Nord, IBHGC—Institut de Biomécanique Humaine Georges Charpak, HESAM Université, 151 Bd de l’Hôpital, 75013 Paris, France; philippe.rouch@epf.fr (P.R.); christophe.sauret@invalides.fr (C.S.); 2 EPF Graduate School of Engineering, 55 Avenue du Président Wilson, 94230 Cachan, France; 3 French Federation of Golf, 68 rue Anatole France, 92300 Levallois-Perret, France; olivier.rouillon@racing92.fr; 4 Racing 92, 11 Avenue Paul Langevin, 92350 Le Plessis-Robinson, France; 5 UF du Centre d’Investigations en Médecine du Sport (CIMS), Hôpital Hôtel Dieu—HUPC, 1bis Place du Parvis Notre Dame, CEDEX 04, 75181 Paris, France; patricia.thoreux@aphp.fr; 6 Université Sorbonne Paris Nord, Arts et Metiers Institute of Technology, IBHGC—Institut de Biomécanique Humaine Georges Charpak, HESAM Université, 151 Bd de l’Hôpital, 75013 Paris, France; 7 Centre d’Etude et de Recherche sur l’Appareillage des Handicapés, Institution Nationale des Invalides, 47 Rue de l’Echat, 94000 Créteil, France

**Keywords:** sport biomechanics, performance, review, golf, recommendation, kinematics, movement analysis

## Abstract

Numerous studies have been conducted to investigate golf swing performance in both preventing injury and injury occurrence. The objective of this review was to describe state-of-the-art golf swing biomechanics, with a specific emphasis on movement kinematics, and when possible, to suggest recommendations for research methodologies. Keywords related to biomechanics and golf swings were used in scientific databases. Only articles that focused on golf-swing kinematics were considered. In this review, 92 articles were considered and categorized into the following domains: X-factor, crunch factor, swing plane and clubhead trajectory, kinematic sequence, and joint angular kinematics. The main subjects of focus were male golfers. Performance parameters were searched for, but the lack of methodological consensus prevented generalization of the results and led to contradictory results. Currently, three-dimensional approaches are commonly used for joint angular kinematic investigations. However, recommendations by the International Society of Biomechanics are rarely considered.

## 1. Introduction

Golf is a widely practiced sport, with approximately 55 million regular players worldwide [1]. In addition to the pleasure of playing, golf has also recognized health benefits. Indeed, it has been shown that practicing golf improves mental and physical health [2]. McHardy et al. [3] highlighted that golf swings are movements that present an injury risk. However, it has also been shown that golf may induce around one injury or experience of pain per five hundred hours of practice [4]. Several factors have been described in the literature for understanding performance or injury occurrence, but nevertheless, there appears to be a lack of consensus on the methodologies for computing commonly used factors such as the X-factor [5,6,7] (parameter for pelvic/shoulder girdle dissociation) or kinematic sequence [8] (sequence of the segmental angular velocities).

Many reviews have been published on golf analysis, including seven focused on health matters: three on low-back pain [9,10,11], one on knee injuries [12], two on injuries in general [3,13], and one on the link between health and golf [2]. Another study focused on electromyographic activity (EMG) measurements during the golf swing [14], with the objective of identifying more activated muscle groups. One study focused on a conditioning program [15]. A narrative review investigated the accessibility of golf in the USA [16], in particular considering the “Americans with Disabilities Act”. However, to our knowledge, no review has yet focused on the biomechanical aspects of the golf swing, even though many articles have been published. However, some issues have not yet been settled, especially around the most common parameters, namely the X-factor and kinematic sequence. We assumed that the substantial variation in parameter estimates may be explained by the different methodologies used.

Thus, the objective of this systematic review was to present state-of-the-art golf-swing biomechanics with a special emphasis on kinematics. When a methodological consensus was reached, the data were extracted. Otherwise, focus was placed on methodological limitations and differences. We then formulated recommendations regarding the methodologies for future studies.

## 2. Materials and Methods

### 2.1. Search Strategy

The methodology used for this systematic review was based on Arksey et al. and Levac et al. [17,18], and PRISMA recommendations [19]. This method comprises the following five steps.

• Step 1: Identification of the research question formulated as “How to describe the biomechanics of the golf swing to explain swing performance or injury occurrence?”

• Step 2: Identification of relevant studies: This step was designed to define the inclusion and exclusion criteria. The inclusion criteria were as follows:

- Studies on golf swing biomechanics;
- Population: all ages, both sexes, all golf skills (recreational, elite, and professional);
- Articles in indexed scientific journals; in case of doubt, the website www.scimagojr.com was used to check;
- Articles available in English only.

After the first request from the Scopus database, the following keywords were identified: golf, swing, biomechanics, kinematics, kinetics, dynamics, angle, velocity, force, moment, GRF, mechanics, power, work, energy, and their variations. A search was conducted on the Scopus, Medline, and IEEE Explore databases on 14 February 2019. Thus, the request was:

Golf AND swing AND (biomechanical biomechanic* OR kinematic* OR kinetic* OR dynamic* OR angle OR velocity* OR speed OR torque OR moment OR force OR GRF OR mechanic* OR power OR work OR energy*).

The search was applied to titles, keywords, and abstracts, and was limited to a timeframe from January 2000 to February 2019.

After evaluating initial results, the following exclusion criteria were defined:

- Articles on other sports (golf only cited as an example but without any specific analysis);
- Master or PhD thesis manuscripts;
- Description and evaluation of commercial devices for golf or equipment testing;
- Analysis of putting;
- Re-conditioning or physical rehabilitation programs without quantitative data on the golf swing;
- Neurologic aspect of the swing;
- Injury studies without reported biomechanical parameters;
- Muscular activation by EMG;
- Articles with only the abstract available and articles not in English;
- Articles without any kinematics results.

• Step 3: Articles were selected based on titles, abstracts, and exclusion and inclusion criteria. Duplicates were removed. If there were any doubts, the article was read. To improve the quality of selection, this step was performed in parallel by two biomechanical experts, and differences were discussed to reach a final decision.

Neal [20,21], Cheetham [22], and McLean [23] were added to the list, as they were often referred to by other articles.

• Step 4: Articles were sorted by category. In this paper, the authors only present the results for kinematic parameters. Two experts defined the categories:

- X-factor;
- Crunch factor;
- Swing plane and club head trajectory;
- Kinematic sequence;
- Segmental and joint angular kinematics.

• Step 5: Analysis: Based on the categorization in the fourth step, we described and evaluated the articles.

### 2.2. Presentation of the Results

First, the common parameters and definitions were gathered. Then, for each parameter, the results were presented and discussed in four steps: (1) the rationale of the parameter, (2) the main results with comments, (3) a comment on the methodology with the authors’ recommendations, and (4) typical values of the parameter of interest from at least one other publication.

## 3. Results and Discussion

### 3.1. Publication Selection

**Figure 1:**
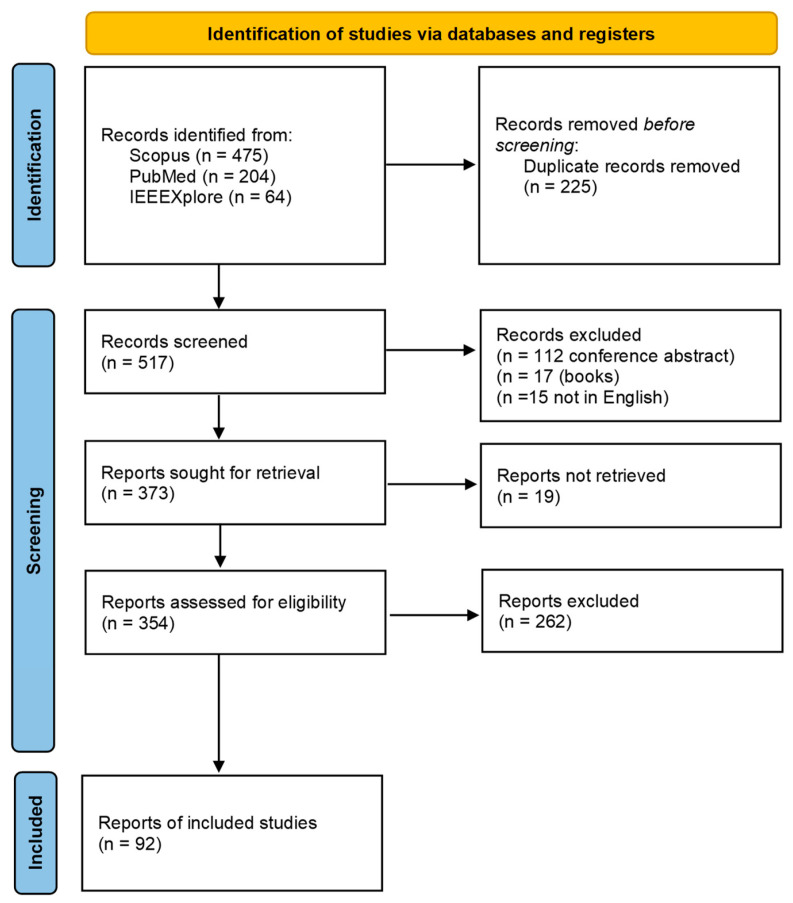
PRISMA workflow.

After removing all duplicates, 517 publications were considered. The application of the exclusion criteria reduced the number of papers to 92, with the publication rate per year increasing from 0 per year in 2000 to 13 in 2018. The PRISMA workflow is given in Figure 1. One limitation of this selection is the use of a database. For example, the Web of Science database was not used. However, regarding overlapping publication bibliographies and databases, the current selection seemed to permit the consideration of a sufficiently large number of publications in the field for performing this review.

### 3.2. Common Parameters

#### 3.2.1. Phases

First, to analyze swing biomechanics, it is necessary to define the phases of the golf swing movement. All studies agreed to define the following four phases:

- The address when the golfer is facing the ball, static and preparing for movement.
- The backswing when the golfer initiates his movement bringing the club up and back.
- The downswing when the golfer accelerates the club forward and downward until it hits the ball.
- The follow-through starts just after the ball impacts the club and aims to stop the movement, that is, decelerating the club.

**Figure 2:**
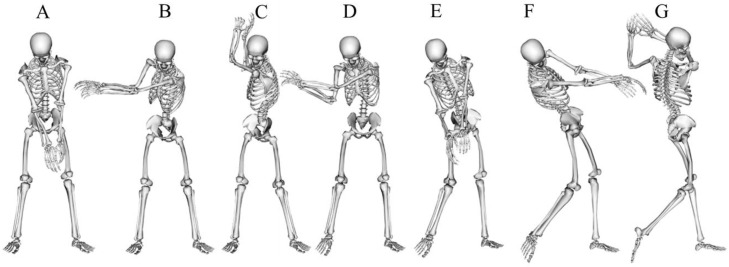
Golf swing sequence [25,26], at different instants: address (**A**), mid-backswing (**B**), top of backswing (**C**), mid-downswing (**D**), impact (**E**), mid-follow-thorough (**F**), finish (**G**).

Some researchers have divided the backswing, downswing, and follow-through phases into two or three sub-phases based on nine events [24] (c.f. Figure 2). Those sub-phases are:

- Take away, corresponding to the initiation of the swing movement.
- Mid-backswing, defined when the club is horizontal during the backswing.
- Late-backswing, defined when the club is vertical during the backswing.
- Top of backswing, defined as the instant when the clubhead speed starts to be oriented downward and frontward.
- Early downswing, defined when the club is vertical during the downswing.
- Mid-downswing, defined when the club is horizontal during the downswing.
- Ball contact or impact, defined when the clubhead hits the ball.
- Mid-follow-through, defined as when the club is horizontal during follow-through.
- Finish, defined as the end of the movement, generally with the club up and back.

These phase detections were based on the club position [26], qualitatively assessed through videos [24], or based on segment positions [20,27]. Recently, Sim et al. [28] compared different methods for accurately estimating the transition instant between backswing and downswing and recommended the use of the vector coding technique (VCT) [29] based on the relationships between several joint angles.

Some studies have focused on phase durations and, more specifically, on the downswing, which is considered to be the most critical phase for performance. The typical values are listed in Table 1. Downswing durations were highly reproducible, with a standard deviation less than 0.04 s for men and 0.08 s for women (there were also fewer studies about women). There was more duration variation between clubs for women than for men, but the average differences remained in the range of the global mean and the standard deviation.

#### 3.2.2. Laterality

Golf swing movement is highly asymmetric. The golfer laterality is defined as:

- **Lead side** or **dominant side**, which is closest to the target. For a right-handed golfer, the lead side is the left side, and vice versa.
- **Trail side** or **non-dominant** side is the farthest side from the target, that is, the right side for right-handed golfers.

### 3.3. Experimental Setup

#### 3.3.1. Rationale

As this review focuses on golf swing kinematics, all articles used experimental data from at least one golfer. However, there were several differences in the experimental setup.

This review emphasizes the measured kinematic data. This section describes and discusses the experimental setup used to measure the data.

#### 3.3.2. Cohort

**Figure 3:**
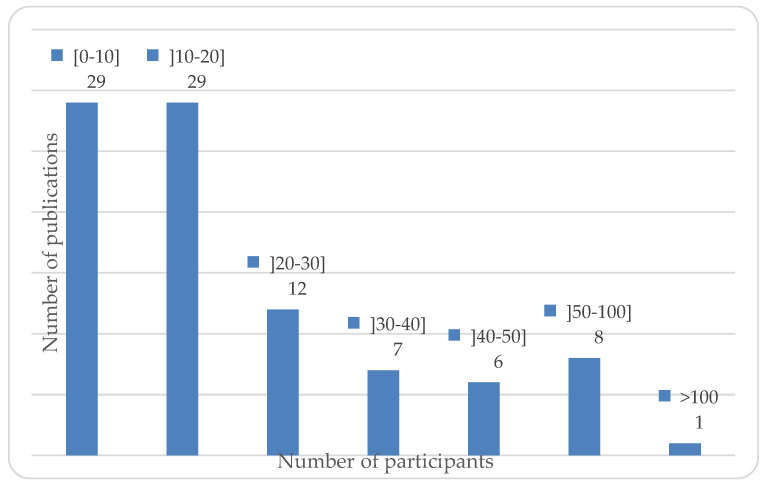
Number of studies with respect to the total number of participants.

Thirty-one articles considered at least one professional golfer. Recreational golfers were often split into two categories: 37 highly skilled (h < 5) golfers and 27 low-skilled golfers; however, two did not provide explicit information about golfers’ skills. The studied groups varied in size from one participant, that is, a case study [34,35,36,37], to a mixed-group analysis of 308 [38]; the majority of studies included between 1 and 20 participants (n = 58/92). The number of participants per publication is shown in Figure 3.

Regarding the studied cohort composition, 65 articles included only men, 1 only women [39] and 23 included both men and women. In addition, 3 articles did not report any information regarding the sex of the volunteers included in the study. In total, 1973 men were included in all the studies, and only 251 women (88.7% versus 11.3%, respectively).

Regarding laterality, 64 articles reported including right-handed golfers, whereas none reported the inclusion of left-handed golfers. However, 28 studies did not report golfer laterality. No golfers were reported to have a swing laterality opposing their hand laterality.

#### 3.3.3. Club

For the majority of the articles, the clubs used were drivers (55 articles), 5-iron (26 articles), 6-iron (5 articles), 7-iron (7 articles), and pitching wedges (3 articles). Sixteen studies used at least two different clubs, and 13 articles did not report any specifications of the club that was used. Two main rationales existed for club influence on swing: either studies compelled golfers to use the same club (six articles), or the golfers were asked to use their own (26 articles).

#### 3.3.4. Performance

The in-field performance of golfers is determined by their golf handicap (*h*). This parameter represents the number of extra shots a golfer needs to carry out to finish a golf course compared to the reference number of shots. Thus, the lower the handicap, the better the golfer. This is a global in-field performance parameter that integrates all the aspects of golf success. However, handicaps are only defined for recreational golfers and not for professional golfers. In addition, as the majority of the studies were carried out in a laboratory and focused on the swing, it was difficult to define the performance with an in-field parameter. Hence, several studies used *h* to characterize their cohort but also gave parameters estimating swing performance during the acquisition.

Only a few studies have investigated swing adaptation to the environment. Blenkinsop et al. [40] measured the adaptation of hip and shoulder alignment with slopes and concluded that there was no significant change with the orientation of the slope.

The majority of the publications investigated how to increase golf swing performance (52/92 articles). In addition, nine studies investigated how to increase a parameter classically considered as a performance criterion without explicitly defining it, such as clubhead speed (seven articles), kinematic sequence (one article), or a comparison of professional versus recreational golfers (one article). In total, 42 studies investigated the speed of the ball (13 articles), or of the clubhead (34 articles), or both (6 articles). Twelve studies compared recreational groups versus professionals without choosing a specific performance parameter, and six articles considered clubhead or ball trajectory angle as performance indicators.

Clubhead and ball speeds and trajectories were measured with a dedicated radar, such as Trackman (TrackMan A/S, Denmark) or Foresight (Foresight Sports, USA). Recently, these technologies were evaluated using high-speed cameras by Leach et al. [41], who suggested that the ball velocity, launch angle, launch direction, spin rate, clubhead velocity, attack angle, club direction, face angle, and dynamic loft can be measured accurately for research purposes with these dedicated radars [41]. However, it should be noted that the ball flight characteristics depend on the coefficient of restitution (i.e., the smash factor). Nevertheless, as it depends on both the clubhead and ball materials and on the golfer technique (all involved in the contact characteristics between the ball and the clubhead), this hinders the comparison of the results between the studies. Typical values of the clubhead speed at impact are listed in Table 2.

Moreover, based on a cohort of 45 men aged between 18 and 80 years with a golf handicap ranging between 2 and 27, Fradkin et al. [42] reported a relationship between clubhead speed at impact and golf handicap, as follows:

$$Club\;head\;speed=e^{4.065-0.0214\cdot handicap}$$

#### 3.3.5. Kinematic Measurement Technologies

The technologies used were mainly based on optoelectronic systems (67 articles), digital videos (9 articles), electromagnetic devices (8 articles), X-rays (3 articles), electrogoniometers (3 articles), and self-produced sensors based on accelerometers and gyroscopes (one article).

**Figure 4:**
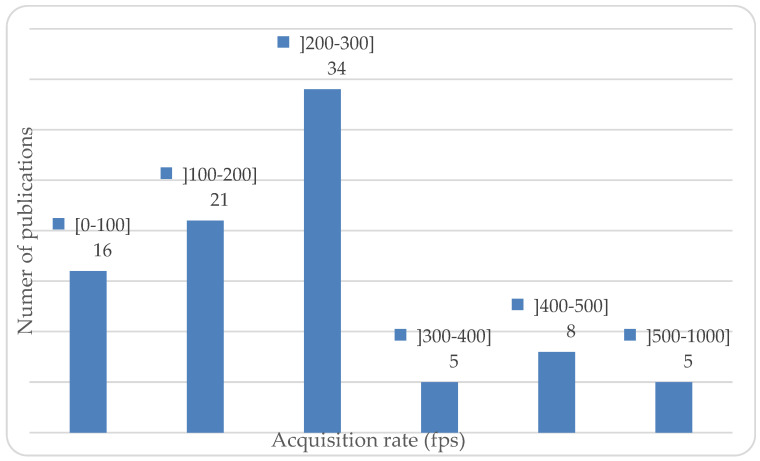
Number of publications for given acquisition rate (in fps) for movement analysis.

The acquisition frequency varied from 3 to 1000 frames per second (fps). The lowest frequencies were observed in three studies using X-ray technologies (3–10 fps) with a very limited number of acquired values. The majority of the studies (55 articles) reported acquisition frequencies ranging between 100 and 300 fps, but three studies did not provide any information about this. A histogram of the acquisition frequencies is shown in Figure 4.

The use of a motion-capture system based on maker tracking is the gold standard in motion analysis. The International Society of Biomechanics (ISB) created recommendations for standardizing marker positioning [49,50,51,52]. However, only 10 articles cited at least one of those articles. Many authors seem to be unfamiliar with soft tissue artifacts [53], as they do not always place the markers on the skin but on suits or clothes.

#### 3.3.6. Recommendations

Most studies have focused on men, were performed in a laboratory, and used an optoelectronic measurement system at a rate ranging from 100 to 300 fps. The participants used a driver club, and the clubhead speed at impact was chosen as the performance indicator. There appears to be a consensus to study clubhead speed at impact or ball speed immediately after impact as a performance indicator for indoor measurements. Golf swing duration appeared to be reproducible regardless of the golfers’ skill, and especially the downswing, which lasts about 0.3 s. Authors would like to highlight that acquisition frequencies for the duration should be adapted. To date, the most frequent acquisition rate is approximately 200 fps. A higher rate would be beneficial, but potentially at the expense of a decrease in the marker tracking accuracy on 2D images. Specific studies using different systems should be performed to determine the best tradeoff between the system frequency and marker location accuracy. The use of ISB recommendations would be beneficial to enable comparisons between studies.

In summary, the articles included focused mainly on right-handed men. Studies on left-handed golfers and women are lacking. In particular, the few studies comparing men and women showed differences; thus, the authors highly recommend filling the gap in knowledge and investigating sex differences for swing analysis.

### 3.4. X-Factor

#### 3.4.1. Rationale

The X-factor was the most common factor described in the scientific literature (31 articles). It was first introduced by McLean [23] and aims to describe the dissociation between the scapular and pelvic girdles during the transition between the backswing and downswing phases. He illustrated it with two lines: one through the shoulders (through both acromia) and one through the pelvis (through the antero-superior iliac spine, on right and left processes) and then defined the X-factor as the angle between the projections of those lines in the horizontal plane where those lines create an “X”. This factor is believed to be linked to performance (a larger X-factor leads to better performance). Basically, an increase in the X-factor is considered as an increase in the shoulder/pelvis dissociation, meaning an increase in the axial rotation of the torso and the shoulder girdle, and thus, an increase in the elastic potential energy of the trunk muscles [54]. Cheetham et al. [55] introduced the X-factor stretch, which is the same factor but computed at the beginning of the downswing and not at the transition between backswing and downswing. This X-factor would be higher for golfers beginning their downswing by rotating their pelvis.

#### 3.4.2. Commentary on the Results

Three studies have investigated the effect of the methodology used on the X-factor values [6,7,34]. Brown et al. [6] considered three different definitions for torso rotation with respect to the pelvis. Kwon et al. [7] also computed the X-factor using three other methods: two considering the shoulder versus the pelvis and one considering the torso versus the pelvis. However, in the latter method, the torso reference frame was expressed with acromia; therefore, this definition is actually a shoulder-versus-pelvis definition. Kwon et al. [7] computed three methods based on shoulder/pelvis dissociation, whereas Brown et al. [6] computed three methods based on torso/pelvis dissociation. Maximal values for Kwon et al. [7] were approximately 60° and the ones for Brown et al. [6] were approximately 30°. This difference is in accordance with a preliminary study [56] based on stereo-radiographs of a participant with a torso axial rotation position, where shoulder-versus-torso mobility contributed approximately 40% of the total axial rotation of the shoulders with respect to the pelvis. Joyce et al. [34] compared the two types of X-factors (shoulders/pelvis and torso/pelvis) for six different orders of rotation for Cardan angle identification and concluded that the best order is (1) lateral bending, (2) flexion/extension, and (3) axial rotation. Thus, Joyce et al. [34] and Brown et al. [6] agreed on the last angle to consider, but not on the first one. In this manner, the axial rotation angle, which is the most pertinent one, is at a position where it is expressed in the distal segment reference frame.

From a methodological point of view, it appears that two main approaches exist for computing the X-factor. The first is strictly linked to McLean’s [23] definition, taking into account one line on the acromia and one line on the anterior part of the pelvis [6,7,34,38,57,58,59,60,61], that is, taking into account the torso and shoulders. The second is focused only on the torso rotation relative to the pelvis. The anatomical landmarks of the torso that were considered in this case were the manubrium, xyphoïd process, 7th cervical spinous process, and 10th thoracic spinous process [31,34,55,62,63]. This choice is essential because the values for the torso-versus-pelvis method are around 30° and values for shoulder-versus-pelvis method are around 60°. De facto, this choice appears to be the main source of variation among studies. The other source of variation is related to the manner of describing the angle: in 3D with a sequence or in projection into a plane (horizontal plane or swing plane).

Another aspect of the definition of the X-factor is temporality. Initially, McLean [23] defined this as the top-of-backswing. However, at the beginning of the downswing, the golfer begins to move with the hips rotating the pelvis. This rotation occurs when the torso is fixed or still rotates in the opposite direction of the pelvis, which favors stretch-shortening cycle involvement of the torso muscles. This means that the maximum value of the dissociation is reached just after the top of the backswing, when the downswing has already begun, not at the of the top of the backswing. For this reason, Cheetham et al. [55] defined the X-factor stretch by analyzing the maximal value of the X-factor at the beginning of the downswing phase, which occurred approximately 1 to 18% after the conventional X-factor. To date, most studies have considered the evolution of the X-factor during downswing. Only Meister et al. [64] also computed an X-factor at impact (shoulders/pelvis) and showed that it was more correlated to performance than its maximum (0.943 vs. 0.900) with iron-5. Finally, some authors have computed the time differentiation of the X-factor during swing [7,31,38,60], to consider the stretching speed of torso muscles. However, as angles were computed differently (sometimes based on the projection in a plane, sometimes from the decomposition in Euler–Bryant or Cardan angles), comparing these results could be difficult. Steele et al. [65] computed the increasing rate of the X-factor and highlighted that the deceleration during the follow through was higher in amplitude than the acceleration during the downswing, particularly for professional golfers.

To date, some studies have reported a link between the X-factor and clubhead speed at impact [38,55,59,60]. However, others have not found a relationship [7,58]. Studies focusing on sex comparisons have shown that women have a smaller dissociation between the torso and pelvis than men [31]. Skill-based comparison showed a difference of approximately 11% for professional golfers compared to recreational golfers. Warm-up was not linked to an increase in X-factor [63]. Nevertheless, recently, Sorbie et al. [66] demonstrated that performing a practice session of 100 swings increased the X-factor and X-factor stretch. However, the population studied by Sorbie et al. [66] was composed of low-handicap golfers (3.3 ± 1.7) able to produce an X-factor of about 50 degrees, contrary to Henry’s participants (15.2 ± 6.7), able to produce an X-factor of about 30 degrees. Thus, warm-up seems to help golfers reach their maximal X factor. Sorbie et al. [67] also investigated the influence of yoga training on golf swing parameters and found a significant increase in the X-factor [67]. Some authors, for example Dale et al. [62] and Joyce et al. [34], have investigated the potential link between X-factor and injury occurrence, particularly low-back pain. Dale et al. [62] suggested that performing partial swing by reducing the backswing amplitude could decrease the compression load on the lumbar spine for golfers suffering from low-back pain, while limiting the decrease in swing performance to approximately 10 m of carry or 2 m/s of clubhead speed. Lamb et al. [68] showed that there was no significant modification of the X-factor when using iron-5 or iron-6, but there was one for X-factor-stretch. Gould et al. [69] showed that golfers with a higher result in the movement competency screening program named “Golf Movement Screen” had an increase in the X factor. They explained their results by improved spine control.

Some authors evaluated the repeatability of X-factor measurement and showed that marker location errors result in a significant change [57]. This questions the relevance of the comparison between studies, as the experimenters are different. Meister et al. [64] compared golf factors between professional golfers and recreational golfers and found a difference in the X-factor of up to two standard deviations for amateurs compared to professional golfers.

#### 3.4.3. Methodological Recommendations

Currently, there is no consensus regarding the recommended methodology for computing the X-factor. This is critical because, depending on the methodology, the results may describe the rotation of the spine or both the spine and the shoulder, leading to different values.

In addition, the authors performed preliminary studies [5,56] that highlighted the following points:

- The plane of projection was not crucial;
- The segment used to compute the X-factor is essential.

Based on the reviewed articles and those preliminary studies, the authors recommend:

If angles are computed directly with two lines, it is suggested to define:

- The landmarks that were used (particularly to distinguish whether the landmarks belonged to the torso or shoulders).
- The plane of projection (which were mainly horizontal plane or swing plane)

If angles are computed from a multibody analysis, the authors should clearly define the segments that are used, the definition of their respective reference frames, and the order of rotation angles that were chosen. The authors are also advised to follow the recommendations of the ISB [50,51] for movement analysis standardization and marker locations.

Finally, the authors recommend clearly indicating the instant at which the X factor is calculated.

#### 3.4.4. Typical Values

Quantitatively, values for the torso-versus-pelvis method are approximately 30°, and those for the shoulder-versus-pelvis method are approximately 60°. Typical values for X-factors are listed in Table 3. 

### 3.5. Crunch Factor

#### 3.5.1. Rationale

The second parameter commonly studied is the crunch factor. It was first introduced by the American Orthopedic Society of Sports Medicine [70]. It was defined as the product of the lateral inclination angle of the torso and the speed of the axial rotation of the torso with respect to the pelvis. The objective of this parameter is to consider both the inclination and axial rotation of the torso that may produce bending stress and shear stress within the intervertebral discs, respectively. These two sources of stress may combine and thus increase stress within the intervertebral disc. The axial rotation speed was considered to determine the loading speed within the vertebrae. Therefore, it attempts to consider their viscoelastic behavior [71] as a combination of axial torque with repetitive flexion/extension motion, which has already been shown to favor hernia occurrence [72].

#### 3.5.2. Commentary on the Results

Lindsay et al., Cole et al., and Joyce et al. [73,74,75] reported no correlation between the crunch factor and the risk of lumbar injury. In addition, there is no consensus on the computation of the X-factor, or more precisely, on how to obtain the torso lateral bending and the speed of torso axial rotation. Ferdinands et al. [58] studied three computation methodologies based only on angular speeds and not on joint angles. However, they did not relate the results to injury occurrences. One study [76] investigated the crunch factor as a performance factor and showed that it was slightly negatively correlated with clubhead speed.

As low-back pain is the most common injury for golf players [3,10], and is, at least partly, linked to disc degeneration [77,78], the crunch factor could help to study the occurrence of low-back pain. However, to date, no study has demonstrated a link between crunch factors and low-back pain. It has been shown that the intervertebral disc is more likely to be injured when loaded cyclically [79] (approximately 10,000 cycles at 0.33 Hz) or by shock. Additionally, it was demonstrated by in vitro experiments that vertebral body or articular facets may be damaged before the disc. Thus, it is difficult to investigate the influence of a factor on injury occurrence, and only a posteriori diagnostic study has been conducted to date.

From a methodological point of view, it appears that there is currently no consensus on the crunch factor; to date, five studies have used 10 different computational methodologies. In particular, Lindsay et al. (2002) [80] indicated values in rad·s^−1^, which appears to be a problem of units, as the correct unit is rad^2^·s^−1^.

#### 3.5.3. Methodological Recommendations

From the authors’ point of view, the only recommendation that can currently be drawn is to explicitly report how the parameters (torso angles and velocity) are computed. Based on the initial definition, the crunch factor should be the product of the inclination angle and axial rotation speed; thus, in rad^2^·s^−1^.

#### 3.5.4. Typical Values

Because there is no consensus on the definition of the crunch factor, Table 4 contains examples using several definitions. The computational method and corresponding values are presented in this table by club type.

### 3.6. Swing Plane and Clubhead Trajectory

#### 3.6.1. Rationale

To describe the swing movement, some authors have limited their study to 2D in the swing plane. They considered the shoulders, arms, hands, and club movements. These segments move roughly within the same plane during the downswing, named the functional swing plane [81]. This approach allowed the development of simple models such as the double pendulum [82], rotational spring [54], and triple pendulum [83,84]. These models were improved, making it possible to perform forward dynamics simulations to optimize the speed and orientation of the clubhead at impact [85]. In these models, the torso rotated around a fixed axis perpendicular to the swing plane, and other segments (generally two: the lead-side arm, and the club) moved within the swing plane.

#### 3.6.2. Commentary on the Results

A swing plane was used to perform simple movement analysis in this plane [54,82,83,84]. However, this concept was questioned by Coleman and Rankin [86], who showed the clubhead to be up to 0.5 m from the swing plane described by the upper limb (shoulder and arm of the leading side). In addition, even if several 2D approaches have been used to analyze the golf swing [54,82,83,84,85], the authors suggest performing 3D analyses to better understand golf swing biomechanics [61].

Different planes were defined and discussed in 17 articles. According to Kwon et al. [81], two main approaches were used for swing plane definition. These were the functional swing plane (defined by the clubhead movement during the downswing) and movement swing plane (defined by points on the shoulder and arm of the leading side). The study by MacKenzie et al. [85] was based on forward dynamics to optimize clubhead orientation at impact, and they indicated the differences between the plane defined with the upper limb and that based on clubhead. They recommended always indicating the definition of the swing plane used in the studies. Recently, Lee et al. [87] computed the functional swing plane to study swing movement using inertial motion unit (IMU) sensors. This plane was also studied by other authors and computed by optimization, either by minimizing the square distance [88,89,90] or by the weighted least square [81] of markers glued on the club during the downswing phase. Nesbit et al. [91] defined two different swing planes: one based on the clubhead and the other based on the hand center. Those planes were at an angle from 9 to 12°.

By considering the swing plane defined by the landmarks of the lead arm, Coleman and Rankin [86] and Coleman and Anderson [88] showed its variation during the downswing. In contrast, Kwon et al. [81] showed good consistency in the plane defined by the club trajectory during the downswing and up to the mid-follow-through for professional players. However, this plane is slightly less consistent for recreational players [81]. This difference between professional golfers and recreational golfers could be linked to the results of Choi et al. [32], who showed a smoother movement (based on jerk analysis) for professional golfers than for recreational golfers. The out-of-plane distance was investigated by Morrison et al. [92] by computing the distance of the actual trajectory compared to its projection in the swing plane during each phase of the swing. The results showed an increase in this distance from address to late backswing and a decrease from early downswing to ball impact. They advised not to compute the swing plane using data during the transition phase (i.e., during top-of-backswing: from late-backswing to early downswing), as they measured a highly non-planar trajectory there.

Club head trajectory was also studied (11 articles). The shape was closer to an eclipse than a circle in the plane [89,93] between the mid-downswing and impact. In addition, ellipse eccentricity was shown to increase with advanced golf skills [89]. Club deflection during the downswing was studied by McGinnis et al. [94], who showed that this deflection occurred mainly within the functional swing plane and was limited to a few centimeters (approximately 5 cm).

The functional swing plane during the downswing is not universal and depends on both the golfer and the club [88,93]. The inclination may be geometrically explained by the length variation of clubs, particularly between drivers and irons. Finally, Sim et al. [95] proposed a new performance parameter based on the computation of the surface area generated by the entire club between two acquisition frames.

#### 3.6.3. Methodological Recommendations

Although different methods were used in the past, there appears to be a consensus to define the functional swing plane during the downswing based on the clubhead position. This plane may be computed as the best-fitting plane as it minimizes the distance from the clubhead trajectory using the least-squares method. The computation should not include the entire downswing. The beginning should be at the early or mid-downswing stage, and the end should be at the impact or the early or mid-follow through stage.

### 3.7. Kinematic Sequence

#### 3.7.1. Rationale

For many different throwing sports, such as javelin throw, handball, and baseball, where the objective is to maximize the velocity of an object at the end of the kinematic chain, the proximal-to-distal activation sequence is considered optimal [96,97,98,99,100]. This sequence is based on the principle of temporal additivity of velocities [20,22,33,57,58,101,102,103]. Thus, the maximum speed at the end of the kinematic chain is obtained for a specific timing of the maximum segmental speeds. The more distal a segment, the later its acceleration should occur. Thus, the higher the number of degrees of freedom to mobilize, the higher the lever arm. For a golf swing, this sequence is often considered optimal for maximizing the clubhead speed at impact.

#### 3.7.2. Commentary on the Results

For golf, a proximal-to-distal kinematic sequence was also defined in the literature (nine articles). This sequence is based on the rotational maxima of the segments of golfers during the downswing phase from the pelvis, torso, shoulder girdles, arms, hands, and finally, the club. Even though the majority of the studies found a higher angular speed for distal segments, the proximal-to-distal kinematic sequence has rarely been verified. Cheetham et al. [22] and Tinmark et al. [33] measured this sequence for professional and skilled amateur golfers by considering either the pelvis, torso, arm, and club segments [22] or the pelvis, torso, and hand [23]. However, this trend was not observed by other authors for recreational golfers and highly skilled amateurs [20,101,102]. Ferdinands et al. [58] also did not observe this sequence, but they focused on the trail side instead of the lead side. In a study based on the forward dynamics method, MacKenzie et al. [104] simulated an optimal swing movement during downswing and confirmed the existence of this optimal kinematic sequence. However, in their simulation, they tested a single participant, who was modeled with only three segments: torso, lead arm (upper arm + forearm), and lead hand + club.

However, the computing modalities for the rotational speeds were different between the studies. Two studies computed the time derivatives of the Euler parameters [21,58], and the latter computed the segmental velocities. The other two computed the angular velocities from the time differentiation of the rotation matrices [33,101]. One study used the instantaneous screw axis theory [102]. One study used a Poisson equation solution and adopted its norm [46]. Cheetham et al. [22] used the time derivative of the axial rotation for the pelvis and torso, and the time derivative of the rotation for the arm and club in the swing plane.

To conclude, seven articles were considered for kinematic sequence investigation. This concept is well-accepted for maximizing the clubhead speed at impact. However, the capacity of golfers, even professional golfers, to perform an ideal kinematic sequence is clearly difficult to realize and measure. According to Neal et al. [21], golfers would be more sensitive to ball–club contact quality than to timing during the downswing. Actually, this timing differs only a few milliseconds as the duration of the whole downswing is 0.3 s [30,31]. For instance, Neal et al. [20] presented timing differences between 4ms and 56 ms, but these values may be very close or below the measurement accuracy (30 Hz in their study). Furthermore, the movement complexity and high number of degrees of freedom to mobilize are some of the sources of bias that the golfer should manage. This may explain why Nesbit et al. [91] measured high interindividual differences in swing kinematics. Finally, a consensus should be reached for the computation methodology, as there is a high variability for velocity computation, for the speed part to be considered, and the segment that should be considered.

#### 3.7.3. Methodological Recommendations

The authors have already shown in a previous publication [8] that the sequence is highly dependent on the computational methods used, and the current technologies are not sufficiently accurate to compute this sequence. In fact, the authors showed that with the same acquisition, the choice of the method used strongly modifies the estimated kinematic sequence. Thus, caution should be exercised when computing the kinematic sequence until a methodological consensus is found, including the measurement protocol limiting soft tissue artifacts [105], optimal acquisition rate, data preparation (smoothing/filtering procedure), and vector component selection.

### 3.8. Joint Angular Kinematics

#### 3.8.1. Rationale

The movements can be described using two different methods. The first relies on the direct use of experimental marker trajectories to define the segment reference frame in space and estimate the angles between the segment reference frames. The second method uses a multibody kinematic optimization technique [106]. The first is easier to implement but is more influenced by marker occlusion and soft tissue artifacts. The second permits the consideration of joint constraints to describe more physiological movements [107]. Recently, Mahadas et al. [108] highlighted the usefulness of OpenSim [109,110], an open-source software based on this methodology, for golf swing analysis.

Estimating joint kinematics involves computing the relative motion between the segments. This permits a description of the movement regardless of the measurement coordinate system. This approach simplifies the description of motion as angles, which are given according to anatomical degrees of freedom. For instance, one would prefer to describe elbow flexion or pronosupination rather than the absolute positions of the arm and forearm within the laboratory measurement coordinate system. To simplify the analysis, the ISB has provided recommendations for defining anatomical frames for segments and joints [49,50,51,52].

#### 3.8.2. Commentary on the Results

This section is mainly focused on joint angular kinematics; tables with typical values for each joint are provided after the commentaries on the results.

##### **Ankle** 

No publication has provided information on ankle kinematics during golf swing with the movement analysis standard of the ISB [49,50,51,52].

##### **Knees** 

Twelve studies considered the knee joint, and only four reported joint angles [47,111,112,113]. Several studies have focused on knee dynamics without providing results on knee kinematics. Murakami et al. [112] performed a reference analysis by creating a three-dimensional (3D) model with a scanner and then performing bone tracking with X-ray images (with 3D model adjustment). This approach is theoretically more accurate, but they only considered six instants (address, early backswing, late backswing, top-of-the-backswing, impact, and end of the follow-through). These values are reported in Table 4. The authors measured a cohort of five recreational golfers, and they can be considered as reference values for studies using optoelectronic motion capture systems, which measured similar values [47,111,113]. Somjarod et al. [113] studied professional and recreational golfers and measured a higher flexion for professionals of approximately 3° at the top-of-backswing (25° vs. 29°). Egret et al. [47] measured a lower flexion of approximately 20° in women compared to men (16 ± 6° vs. 35 ± 5°).

Internal–external rotation of the knee has also been measured by Murakami et al. [112]. For the leading side, the global amplitude ranged from −7° to 10°, whereas it varied from −16° to 10° for the trail side.

Abduction–adduction kinematics of the knee were investigated by Kim et al. [114,115], who aimed to demonstrate the effectiveness of using a lateral heel wedge to reduce knee pain or anterior cruciate ligament rupture. They measured angles between 0.42 ± 0.73° and 5.95 ± 2.91° without the wedge club and between 0.30 ± 0.86° and 5.99 ± 3.17° with a wedge. They concluded that the wedge may reduce varus moment, but they did not show any results in terms of joint dynamics. However, these values were very similar, highlighting a trend.

Although Murakami et al. [112] used two-dimensional (2D) scanner images, they estimated an accuracy of 0.3° for the rotation. However, the article by Ishimaru et al. [116], presented as the reference for method accuracy, studied patellar movement. The validation of the rotation accuracy seemed to be only performed for elementary movements with a lower acquisition frequency (three versus ten images per second). The study focused on elderly patients who underwent knee arthroplasty, and validation was performed on pig cadavers. Thus, one may expect lower accuracy for this more complex movement. This could explain the differences between the scanner image method and the optoelectronic method. However, the study of Murakami et al. [112] was the only one able to measure the antero-posterior translation during movement: 4.6 ± 9.2 mm for the lead side and 4.1 ± 3.6 mm for the trail side.

The knee kinematics of recreational golfers have been shown to differ from those of professional golfers. Kim et al. [117] highlighted that professional golfers flexed their trail knee less, and Choi et al. [32,111], measured a second peak for a golfer lead knee. In contrast, Somjarod et al. [113] did not find any significant differences in trail knee flexion between professional and recreational golfers. Somjarod et al. [113]. also measured internal–external rotation of the knee, but their values were different between professional (−20° at the top of backswing) and recreational (−26° at the top of backswing) golfers. However, even though they presented values for the knee, these values appeared closer to the hip values. The method used was not well-detailed in the article, making it difficult to analyze the data, especially because the link between hip and knee internal–external rotations remains unknown.

Finally, Purevsuren et al. [118] investigated the link between anterior cruciate ligament injury risk and knee kinematics. They highlighted the increase in ACL loading with decreased knee flexion and increased tibial rotation [118].

##### **Hips** 

Eleven studies considered hip kinematics [32,47,112,117,119,120,121,122,123,124] and eight articles reported joint angles [47,117,119,120,121,122,124]. One publication [112] reported values based on femur movement without the pelvis, which is needed to create the hip joint frame. Only two studies [32,117] decomposed the hip angle into its three basic components, but only the study by Kim et al. [117] reported hip joint kinematic values. The values are listed in Table 4. Other studies have provided superior values for hip angles. It was shown that hip movements were highly asymmetric [121], and a higher internal–external range of motion was observed for the lead hip than for the trail hip. The lead hip used almost the entire physiological range of motion of the hip in external rotation, backswing, and internal rotation during the downswing. This was confirmed by Alderslade et al. [119], who measured the hip internal–external rotation during the swing that remained within the passive angular corridor.

In addition, lead hip movement was found to be highly linked to torso movement and was positively correlated with clubhead speed at ball impact [120]. Mun et al. [124] showed that rotation was initiated by the lead hip, followed by the lumbar spine; for professional golfers, lumbar and lead hip rotations were equally distributed. A lack of mobility for the lead hip has been linked to higher use of the lumbar spine [117]. This could explain the efficiency of a hip-stretching program in limiting low-back pain occurrence when golfers lack hip mobility [125]. Finally, Egret et al. [47] highlighted the differences between women and men, with higher hip movement amplitudes for women.

Finally, one publication [126] investigated the joint angle differences induced by slight modifications to the ball position at the address. With the ball position varying by 4.3 cm, the hip flexion was modified up to 1.5° relative to the reference position. However, the mean variation was within the standard deviation of the reference frame, and the authors only considered the flexion–extension of the hips.

##### **Torso** 

Torso kinematics during golf swings have often been studied. Some authors included more details than on the X-factor. There were three different approaches: injury prevention, performance improvement, and group difference investigation.

To date, two studies have focused on the modern swing, which is characterized by the need for a higher axial rotation of the torso. They suggested that the modern swing was associated with a higher injury risk in the lumbar spine [62,127]. However, Lindsay et al. [75] did not measure any significant kinematic differences between asymptomatic players and players with low-back pain, using a driver. Kim et al. [117] found that a lack of hip internal–external rotation was compensated by a modification of the pelvis kinematics, in particular, the posterior tilt and flexion of the lumbar spine.

It was also demonstrated that an increase in torso axial rotation was correlated with an increase in the clubhead speed at ball impact [128], which is the same effect as the X-factor. Okuda et al. and Zheng et al. [48,129] found that skilled golfers began their torso rotation earlier than less-skilled golfers. Chu et al. [38] suggested that flexion/extension and lateral bending of the torso are kinematic parameters involved in performance. Furthermore, Joyce et al. [130] estimated that torso kinematics contributed 34–67% of the performance variance. Two studies identified coupling between torso and pelvis rotations, suggesting that experienced golfers succeeded in modifying their neuronal networks to synchronize their movements. For professional players, Beak et al. [131] found a correlation between torso and pelvis speed peaks.

Sex-related differences were also assessed. On the one hand, Zheng et al. [132] showed that torso rotations were not significantly different between genders. On the other hand, Horan et al. [31] showed that men and women did not have the same optimal swing, and torso and pelvis movements were not the same between sexes.

Lindsay et al. [75] showed that the torso kinematics differed according to the club used. The results differed when using a driver or 7-iron for flexion and lateral bending. Finally, Horan et al. [133] highlighted that performing a putting session before swinging improved torso mobility, specifically for women. This is quite contradictory to Henry et al. [63], who found no effect on the X-factor value of the warm-up before swinging.

One publication [126] investigated the differences induced by slight modifications of the ball position at the address, although they did not directly correlate their findings with performance. The torso side bending and torso flexion were measured in the global frame. Only torso flexion was modified by with a minimal modification of about 1°.

One publication [134] measured the coupling between the pelvis and torso rotation angles and highlighted different patterns depending on golfer skills.

##### **Neck** 

Only three studies considered neck kinematics or head movements [32,46,58]. In particular, Horan et al. [46] presented a new kinematics sequence: head, pelvis, and torso, in terms of rotational speeds for their participants. They measured a speed of approximately 210 ± 56°/s. However, the interest in taking the head for the kinematic sequence remains unclear.

##### **Shoulder** 

The shoulder joints have often been studied. However, the marker sets used were often minimal. The more common marker set (torso: manubrium, xyphoïd, acromions, 7th cervical vertebra, and 10th or 8th thoracic vertebra; arm: lateral and/or medial epicondyles of the humerus) was used to study the glenohumeral joint, with the assumption that the scapular girdles (clavicles and scapulae) were motionless in the torso. Ferdinands et al. [58] measured the global shoulder speed of approximately 6 rad/s. Teu et al. [135]. measured the contribution of each degree of freedom to the clubhead and estimated the internal/external rotation of the arm to contribute 14%, adduction/abduction 12%, and retroversion/anteversion 1%.

Some studies have focused on sex differences and have shown kinematic differences between them. Zheng et al. [132] measured a significant difference in shoulder orientation, defined as the angle of the acromia line relative to the room frame. Egret et al. [47] measured a significant difference between men (82°*) and women (110°*).

Variation induced by skill differences was also investigated. On the one hand, Choi et al. [32] measured no significant difference regarding shoulder kinematic smoothness (based on the jerk computation, the time derivative of acceleration). On the other hand, Healy et al. [136] measured a higher value of right shoulder flexion at the top of the backswing for experienced golfers, with a higher clubhead speed at impact with 5-iron. Egret et al. [30] also showed that experienced players appear to have a larger shoulder angle than less experienced players. Mitchell et al. [137] measured the variation in joint mobility in groups of golfers of various ages. They measured the decrease in shoulder mobility with age. Adduction in the horizontal plane was an exception, with an increase during the backswing. Finally, differences induced by clubs were investigated by Egret et al. [138], who found shoulder kinematic differences between drivers and 5-iron clubs and between drivers and pitching wedges, but not between 5-iron and pitching wedges.

One study [126] investigated the differences induced by slight modifications in the ball position at the address. However, the induced modifications were very small for shoulder kinematics (less than 1° between configurations) and not statistically significant.

Finally, one publication [25] addressed the issue of the negative effect of using a rough model for golf swing kinematic processing. They showed that even if the glenohumeral joint was the only one considered for golf swing analysis, the scapulothoracic and thoracoclavicular joints are used during the golf swing. Consequently, an inaccurate model of the shoulder joint may lead to inaccuracies in neighboring segments. Furthermore, they also published the values of inverse kinematics during the golf swing.

##### **Elbow** 

Even though elbows are often studied, their role in performance remains unknown. Only Zheng et al. [48,132], and Egret et al. [47] highlighted a kinematic difference; the more skilled the players are, the more able they are to extend their elbow during the swing. Additionally, according to Egret et al. [47], professional women seemed to have a faster elbow extension than professional men. They also measured [47] a higher amplitude for women than men, with a smaller angle at the top of the backswing and a higher angle at impact, which was in agreement with Zheng et al. [132]. From an injury point of view, McHardy et al. [3] showed that recreational golfers and women were more likely to have an elbow injury than professional golfers and men, respectively.

##### **Wrist** 

Several studies have indicated a positive correlation between wrist movements and performance [48,59,91,128,135,139,140,141]. The wrist deviation angle was shown to be higher for skilled amateur golfers or professional golfers than for high-handicap recreational golfers [140]. They also tended to unlock their wrist [38,139]. However, the marker set used by Chu et al. [38] was limited, with only two markers for the forearm and the wrist (one on the lateral epicondyle of the humerus and one called “wrist” without more detail) and two on the club shaft. Even if no modeling and computation details were provided, the results were in accordance with those of Betzler et al. [139].

Using a dynamic model of the club and upper limb, Suzuki et al. [141] found that a late wrist release movement increases the clubhead velocity. Thus, as the wrist is at the end of the kinematic chain, its movement seems to amplify the velocity production just before impact and has a reduced mass moment of inertia during the first part of the downswing by placing the club and the upper segment close to the axial body. Regarding typical values for wrist kinematics, Zheng et al. [48] measured an amplitudes 45° (trail side) and 70° (leading side) for wrist angle. Their marker set was quite minimalist, as they appeared to have only one marker per hand, but they defined wrist movements based on forearm movements relative to the club.

Another study investigated the effect of grip material on wrist kinematics [142]. They measured wrist kinematics for three degrees of freedom (flexion–extension, radioulnar deviation, and internal–external rotation) on 12 PGA coaches. They showed that strong, neutral, and weak grip lead to the same clubhead velocity at impact, but its right/left orientation angle was different from −1.5 ± 4.7° (strong), −2.6 ± 4.5° (neutral) to −6.4 ± 6.9° (weak).

Sorbie et al. [67] measured hand speed during the downswing before and after a yoga training program and showed a slight improvement of approximately 2 m/s for a hand speed of 30 m/s.

Finally, Todd et al. [143] investigated whether a partial swing is a scaling of a full swing. They found that the wrist angle was higher for a partial swing than its theoretically scaled value [143]. This angle was defined between the forearm and club shaft.

#### 3.8.3. Methodological Recommendations

Considering the results presented in this section, joint angular kinematics has attracted significant interest. The main anatomical degrees of freedom were measured. Although some 2D approaches have provided reasonable results in the past, it appears more appropriate to use a 3D approach. Unfortunately, the methodological details are often insufficient. Thus, it is difficult to reproduce or aggregate the results of many studies. Few studies have investigated joint angle kinematics based on ISB recommendations for anatomical frame and angle definitions [49,50,51,52]; those recommendations could be a means for harmonizing joint angular kinematic data. Marker sets were often minimalist and seemed to only measure global kinematic behavior. Moreover, as highlighted by Mears et al. [144], interactions between measured degrees of freedom may help understand and advise on golf swing techniques.

The ISB has provided some recommendations for their definitions [49,50,51,52], and the authors would encourage following these recommendations in future studies.

#### 3.8.4. Typical Values

In the following tables, typical values are given for each degree of freedom and for the movement amplitude during the entire swing. One study was selected to illustrate the results for each degree of freedom.

##### **Ankle** 

No publication has reported joint angles of the ankle during a golf swing with a movement analysis standard.

##### **Knees** 

Murakami et al. [112] performed an analysis based on X-ray images, which can be assumed to be more accurate. These are listed in the following Table 5:

In time evolution, the knee flexion angle for the leading side was 18 ± 12° at the address, 22°* at the early backswing, 26°* at the late backswing, 33 ± 8° at the top of backswing, 25°* at impact, and 16 ± 9° at the end of the follow-through. For the trail side: 17 ± 9° at the address, 18°* at the early backswing, 23°* at the late backswing, 24 ± 8° at the top of backswing, 22°* at impact, and 19 ± 6° at the end of the follow through. They also measured internal–external rotation of the knee for the leading side: 2 ± 6° at the address, −7 ± 7° at the top of backswing, and 10 ± 5° at the end of the follow-through. For the trail side: 1 ± 9° at the address, 10 ± 5° at the top of backswing and −16 ± 5° at the end of the follow-through.

##### **Hips** 

Kim et al. [117] investigated differences between golfers with limited hip rotation and asymptomatic golfers. As they focused their analysis on hip joint kinematics with anatomical angles, their values were used as examples, as shown in Table 6.

##### **Torso** 

Torso kinematics have rarely been fully described in terms of anatomical joint angle kinematics. Bourgain et al. [25] reported values for the three anatomical angles, and they were selected as examples in Table 7.

##### **Neck** 

No publication has reported joint angles of the neck during a golf swing with a movement analysis standard.

##### **Shoulder** 

The shoulders are often limited to the glenohumeral joint. Thus, the study by Bourgain et al. [25] was chosen as an example for Table 8 as their study has a detailed description of shoulder kinematics by describing the glenohumeral, sternoclavicular, and scapulothoracic joints.

##### **Elbow** 

Elbow kinematics have rarely been fully described in terms of anatomical joint angle kinematics. As Bourgain et al. [25] reported values for both flexion and pronosupination angles, they were selected as examples in Table 9.

##### **Wrist** 

Wrist kinematics are rarely fully described in terms of anatomical joint angle. As Bourgain et al. [25] reported values for both flexion and deviation angles, they were selected as examples in Table 10.

## 4. Conclusions and Perspectives

This systematic review highlighted that there is a growing interest in the kinematics of the golf swing. There is a consensus in the definition of movement, with four main phases (address, backswing, downswing, and follow-through). The technologies used mainly consisted of an indoor motion analysis system based on optoelectronic motion capture systems. Until now, study cohorts were mainly composed of recreational golfers, highly skilled amateurs, and professional golfers with an equal distribution. However, these studies mainly focused on right-handed men. Thus, there is a lack of studies on women and left-handed players. Although one could expect a slight change with the dominant side, more importantly, publications comparing men and women highlighted biomechanical differences, which should be analyzed. Thus far, there have been no articles focusing on women only.

Some simple parameters have been proposed to describe the performance or risk of injury. Studies have mainly focused on the X-factor, crunch factor, swing plane, kinematic sequence, and joint angular kinematics. From a methodological point of view, there is limited consensus on the elements. However, even if there is a consensus regarding the rationale of using some parameters (e.g., X-factor, kinematics sequence), the lack of methodological consensus drives variation in measurement and interpretation. Proposing a standardization of methodologies would help to ensure its mechanical trueness and will help players, coaches, and medical staff to trust those approaches and permit the collection of data. A more in-depth investigation of the rationale of these parameters combined with advanced skills in motion analysis methodologies (good knowledge of possibilities and limitations of material and data processing) would allow the public to be provided with such recommendations for standardization. Meanwhile, it would be possible to standardize the expression of the segment and joint kinematics by following the recommendations of the International Society of Biomechanics. Methodologies have rarely been fully described, making it more difficult to check the quality of the methodologies; the word limits in publications may favor this lack of information.

The main limitation of this systematic review was the focus on kinematics. Many studies have been published on kinematics or geometric concerns because they are believed to be easy to understand and compute. However, as shown in this review, these parameters may be more difficult to process or analyze than expected. Thus, a review focusing on kinetics, including ground reaction forces and net joint moment, should also be performed to complete the overview initiated with the present one.

Interest in performance and injury prevention continues to increase. Thus, kinematic analysis of the golf swing continues to search for technologies that permit accurate estimation of golfer kinematics. Recently, embedded technologies based on inertial measurement units or accelerometers have been used for movement analysis. These technologies appear promising for accurate and more ecological measurements than currently used technologies (optoelectronics, electromagnetic, or electrogoniometer systems). In addition, new technologies have opened new possibilities, such as machine-learning algorithms to improve the analysis of videos [145] or machine learning for understanding performance [146]. However, these news tools are complex to develop and understand. For instance, the quality of a machine-learning algorithm is directly correlated to the quality of the dataset, both for training and operation. In addition, these tools are often combined with models that are developed based on assumptions. Thus, the user should be clear on the measurement target and methodology in addition to having good methodological skills to identify limitations in interpretation.

Understanding performance remains complex and requires combining different fields of research with the involvement of athletes, their training, and medical staff. This review may help researchers, trainers, athletes, and medical staff to understand the state-of-the-art golf swing biomechanics. These elements are beneficial for improving knowledge and developing new analysis protocols.

## Figures and Tables

**Table 1:** Typical values of downswing phase duration for the driver and the irons, given in seconds. h means golf handicap. The value source is given in brackets.

Club	Gender	Recreational Golfers (h > 5) (s)	Highly Skilled Amateurs (h < 5) (s)	Professional Golfers (s)
Driver	Male	0.25 ± 0.02 [30]	0.31 ± 0.04 [31]	0.31 ± 0.04 [32]
	Female		0.39 ± 0.08 [31]	
Iron	Male		0.31 ± 0.03 [33]	0.28 ± 0.03 [33]
	Female		0.36 ± 0.06 [33]	

**Table 2:** Typical values for clubhead speed at impact.

		Recreational Golfers	Highly Skilled Amateurs	Professional Golfers
Men	Iron	33.8 ± 2.5 m/s [43]	37.65 ± 1.04 m/s [44] ^a^	
	Driver	[33 *–53 *] m/s [42]	[55 *–57 *] m/s [42] 45.4 ± 3.6 m/s [45]	50.1 ± 2.1 m/s [46]
Women	Iron			
	Driver	37.7 ± 3.8 [47] ^b^		32 ± 1 [48]

^a^ The group of this study is composed of golfers either professional or recreational with an handicap inferior to 1. ^b^ The group of this study has an handicap of 6.1 ± 3.4. * means that the value was extracted from a plot or a chart. The value source is given in brackets.

**Table 3:** Typical values of X-factors (in degrees).

		2D Angle: Horizontal Plane (°)	2D Angle: Swing Plane (°)	3D Angle (°)
Recreational golfers	Torso–pelvis	28 * ± 13 * [6]		28 * ± 13 * [6]
	Shoulders–pelvis	57.1 ± 11.2 [7]	57.7 ± 10.5 [7]	54.4 ± 10.3 [7]
Professional golfers				48 [55] ^a^

The ^a^ group was composed of 8 professional golfers and 2 highly skill golfers with a handicap inferior to 1. * Directly read from a plot or a chart. The value source is given in brackets.

**Table 4:** Typical values of the crunch factor according to the methodology used. The value source is given in brackets.

Publication	Methodology (Parameter1·Parameter2)		Values	
	Parameter1	Parameter2	Driver	Iron
Cole et al. [73]	Axial torso rotation	Lateral bending angle	1.5 rad^2^·s^−1^	
Joyce et al. [76]	Lateral bending (upper torso)	Axial rotation velocity	3.0 ± 0.8 rad^2^·s^−1^	3.0 ± 0.5 rad^2^·s^−1^
	Lateral bending (lower torso)	Axial rotation velocity	0.5 ± 0.2 rad^2^·s^−1^	0.5 ± 0.1 rad^2^·s^−1^
Lindsay et al. [75]	Axial rotation velocity	Side bending angle	with low-back pain: 82.4 ± 21.9 rad·s^−1^	
			without low-back pain: 87.7 ± 28.4 rad·s^−1^	
Ferdinands et al. [58]	Pelvic tilt velocity	Pelvic axial velocity		8 *rad^2^·s^−2^
	Thoracic lateral bending	Pelvic axial velocity		5 *rad^2^·s^−2^
	Thoracic flexion	Pelvic axial velocity		12 *rad^2^·s^−2^
Joyce et al. [74]	Torso lateral bending	Torso axial rotation	2.9 ± 0.6 rad^2^·s^−1^	
	Lower torso lateral bending	Lower torso axial rotation	0.3 ± 0.2 rad^2^·s^−1^	

* Directly read from a plot or a chart. The value source is given in brackets.

**Table 5:** Typical values for knee joint angular kinematics.

Knees [112]	Leading Side	Trail Side
Internal/external rotation (°)	18 *	25 *
Adduction/abduction (°)	Not given	Not given
Flexion/extension (°)	15 *	8 *
Antero-posterior translation (mm)	5	4
Medio-lateral translation	Not provided	Not provided

* Directly read from a plot or a chart. The value source is given in brackets.

**Table 6:** Typical values for hip joint angular kinematics, given in degrees.

Hips [117]	Leading Side	Trail Side
Internal/external rotation (°)	50 *	40 *
Adduction/abduction (°)	45 *	40 *
Flexion/extension (°)	30 *	45 *

* Directly read from a plot or a chart. The value source is given in brackets.

**Table 7:** Typical values for the torso kinematics. Extracted from a participant of the Bourgain et al., 2018 study. The value source is given in brackets.

Torso [25]	Values
Axial rotation (°)	129
Lateral bending (°)	28
Flexion/extension (°)	33

**Table 8:** Typical values for the shoulder kinematics. Extracted from a participant of the Bourgain et al., 2018 study. The value source is given in brackets.

Shoulder [25]	Leading Side	Trail Side
Clavicle protraction (°)	27	38
Clavicle elevation (°)	25	6
Shoulder elevation (°)	100	13
Humeral flexion (°)	42	34
Humeral axial rotation (°)	64	125

**Table 9:** Typical values for the elbow kinematics. Extracted from a participant of the Bourgain et al., 2018 study. The value source is given in brackets.

Elbow [25]	Leading Side	Trail Side
Elbow flexion (°)	26	95
Pronosupination (°)	153	71

**Table 10:** Typical values for the wrist kinematics. Extracted from a participant of the Bourgain et al., 2018 study. The value source is given in brackets.

Wrist [25]	Leading Side	Trail Side
Flexion	38	86
Deviation	90	28
